# uPAR directed-imaging of head-and-neck cancer

**DOI:** 10.18632/oncotarget.16240

**Published:** 2017-03-15

**Authors:** Victor M. Baart,, Martin C. Boonstra, Cornelis F.M. Sier

**Affiliations:** Leiden University Medical Center, Department of Surgery, Albinusdreef, Leiden, The Netherlands

**Keywords:** urokinase receptor, image-guided tumor imaging, monoclonal antibody, peptide, near infrared fluorescence

According to two recently published pre-clinical studies, targeted multimodal imaging via uPAR (urokinase plasminogen activator receptor) could be the next step in achieving more balanced radical resections in head-and-neck cancer surgery [1, 2]. Multimodal imaging, using a single targeting agent conjugated with a radionuclide as well as a near infrared (NIR) fluorescent dye, is able to provide essential information before (radionuclide, PET/SPECT) and during (NIR fluorescence imaging) surgery, allowing sharp delineation between tumor and surrounding tissue. Sharp assessment is especially crucial for head-and-neck cancer surgery, where sparing of healthy tissue can prevent functional loss and improve cosmetic outcome [3]. The expression of uPAR, a key player in tumor cell adhesion, proliferation and migration, in tumor tissue and its absence in normal tissue allows for sub-millimeter delineation of tumor edges and casts it as a robust target for imaging.

Both studies use uPAR recognizing agents with comparable multimodal labels in similar models but differ in the targeting vehicle (Figure): a smart peptide (AE105, 1 kDa) versus a monoclonal antibody (ATN-658, 150 kDa). The nonamer peptide AE105 has been optimized from a 15-mer variant, identified from a random-phage display library. It has a high affinity for the third domain (D3) of uPAR in the uPA-binding cavity (see Figure) [4]. Due to its small size, the imaging timeframes of AE105 are relatively short, generally within several hours [5, 6]. The monoclonal antibody ATN-658 is a mouse IgG antibody (humanized version: huATN-658), binding to the c-terminus of uPAR in the D3 domain. ATN-658 exhibits relatively long serum half-lives (15-20 hours), showing imaging timeframes of up to days [7, 8]. The longer timeframe of antibodies are caused by the larger size rendering them well-suited for multimodal clinical applications, where preoperative PET or SPECT imaging and intraoperative NIR fluorescent imaging presumably take place over a couple of days. In the presented study, AE105 was conjugated separately with each label and administered consecutively. ATN-658 was conjugated with a hybrid label and administered once. For hybrid conjugation the compromise has to be made that none of the labels can be fully optimized, whereas administering multiple labels consecutively can result in *in vivo* competition and allergic reactions. The generally observed and size related hepatic clearance of antibodies is disadvantageous for the detection of liver metastasis, but is not relevant for head and neck cancer [8]. Next to size, the *in vivo* behavior of imaging agents dependents on other physical characteristics like affinity, lipo- or hydrophilicity and net charge, which are influenced by the conjugated dyes and chelators used for radiolabeling especially with small peptides.

Both AE105 and ATN-658 have been designed for anti-tumor activity but achieve this differently. AE105 is a competitive inhibitor of uPA binding to uPAR [4]. On the other hand, ATN-658′s anti-tumor activity is independent of the uPA-uPAR interaction (Figure) and is attributed to its antagonistic effects on integrin-uPAR interactions, possibly leading to disruption of the uPAR signalosome [7]. Since AE105 is incapable of displacing formed uPA-uPAR complexes, it is unable to target uPA occupied uPAR [4]. Consequently, for imaging applications the signal intensity of tumors targeted with AE105-based agents will depend on the degree of uPAR saturation by uPA. ATN-658 binding is not affected by uPAR-occupancy with uPA, possibly leading to a stronger and perhaps more relevant signal (Figure).

**Figure:**
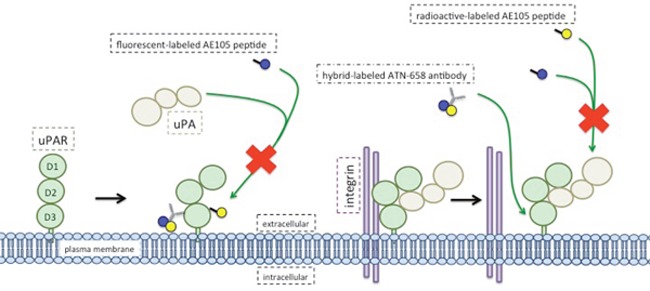


Given that AE105 and ATN-658 are different targeting agents, their production and clinical translation diverge significantly. Humanized antibodies are generally produced in mammalian cells, while peptides are synthesized using non-biological systems, leading to favorable safety profiles and lower costs. However, antibodies are known to possess superior binding characteristics as they exhibit high specificity and long half-life times, are stable and show sufficient tumor penetration. Comparison of both concepts is difficult in animal models. Although ATN658 and AE105 both show encouraging results, the real clinical value of uPAR targeted multimodal imaging will come from clinical trials and associated follow-up studies, that are to be performed within the next years. The first phase 1 trials have recently been published for AE105 [4, 5] and therapeutic trials are expected to begin for the humanized version of ATN-658 in early 2018. Only then will we know whether patients really benefit from enhanced uPAR-based imaging techniques, either by improved quality of life or increased survival. Until then, basic science and pre-clinical research should further widen our understanding of uPAR targeting and explore the possibilities for clinical applications. Possibly, targeting agents with various characteristics might be needed in the clinic: Peptides may be more amenable as single labelled agents in acute situations for direct imaging, whereas antibodies may be useful for more elective applications like oncologic surgery, where both pre-operative imaging as well as intraoperative guidance is desired.
